# Plant-based meat substitute analysis using microextraction with deep eutectic solvent followed by LC-MS/MS to determine acrylamide, 5-hydroxymethylfurfural and furaneol

**DOI:** 10.1007/s00216-023-05107-6

**Published:** 2023-12-21

**Authors:** Dominika Osiecka, Christina Vakh, Patrycja Makoś-Chełstowska, Paweł Kubica

**Affiliations:** 1 Department of Analytical Chemistry, Faculty of Chemistry, Gdańsk University of Technology, 11/12 G. Narutowicza Street, 80-233 Gdańsk, Poland; 2 EcoTech Center, Gdańsk University of Technology, 11/12 G. Narutowicza Street, 80-233 Gdańsk, Poland; 3 Department of Process Engineering and Chemical Technology, Faculty of Chemistry, Gdańsk University of Technology, 11/12 G. Narutowicza Street, 80-233 Gdańsk, Poland

**Keywords:** Plant-based meat substitutes, Natural deep eutectic solvent, Acrylamide, 5-Hydroxymethylfurfural, Furaneol, LC-MS/MS

## Abstract

For the analysis of plant-based meat substitutes and the determination of Maillard reaction products such as acrylamide, 5-hydroxymethylfurfural and furaneol, a novel and effective procedure based on hydrophobic natural deep eutectic solvent and liquid chromatography coupled with tandem mass spectrometry was developed for the first time. The 49 compositions of the deep eutectic solvents were designed and screened to select the most suitable option. The terpenoids eugenol and thymol in a molar ratio of 2:1 were selected as precursors for solvent formation, allowing effective extraction of the target analytes. The developed procedure comprised two main steps: extraction — in which the analytes are isolated from the solid sample due to the salting-out effect and pre-concentrated in the deep eutectic solvent, and back-extraction — in which the analytes are re-extracted into the formic acid solution for subsequent mass spectrometric detection. As the density of the aqueous phases changed during the extraction and back-extraction steps, the phenomenon of inversion of the coalesced organic phase was observed, which simplified the withdrawing of the phases. The linear range was 1–50 ng/mL for acrylamide, 10–1000 ng/mL for 5-hydroxymethylfurfural and 200–1000 ng/mL for furaneol with coefficients of determination above 0.9952. The developed method was fully validated and found recoveries were in the range 83–120%, with CVs not exceeding 4.9%. The method was applied to real sample analysis of pea-based meat substitutes.

**Graphical abstract:**

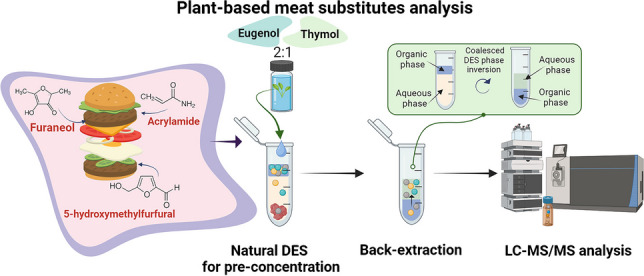

**Supplementary Information:**

The online version contains supplementary material available at 10.1007/s00216-023-05107-6.

## Introduction

At a time when climate change is being discussed, animal husbandry and its impact on the planet are an important issue [1]. Due to the livestock sector, not only is the emission of greenhouse gases major [2], but also the land use and resource consumption needs to be considered [3]. A large-scale dietary shift to a low-meat diet can effectively mitigate climate change and may also have health benefits [4]. Meat substitution by-products intended to be meat-like is an interesting alternative to reduce meat consumption [5]. Although it is predicted that plant-based meat substitutes (PBMS) market will be much less valuable in the next few years compared to the meat market, there is a growing tendency in the launches of new PBMS [6]. Modern plant-based protein food not only includes well-known soy or wheat derivatives such as tofu, tempeh or seitan [7], but also products that are designed to be indistinguishable by taste, feeling, texture and applications with typically meat-based foods [8]. Therefore, PBMS are considered as a new generation of meat-like products and include plant-based burgers, meatballs, chicken-like pieces, etc.

PBMS have completely different quality control requirements than meat-based products [9–11]. In addition, plant proteins need to be texturized to give them meat-like fibrous structure. Shear cells, electrospinning, freeze structuring, extrusion and even 3D printing are most commonly used for this purpose [12]. Among these techniques, extrusion is not only well-developed but also well studied, as it is applied to many food type manufacturing [13]. Since extrusion is a multistage process that involves high temperatures and pressure, the Maillard reaction can occur, leading to the formation of different reaction products depending on the raw materials used [14]. Maillard reaction begins when amino groups in proteins, peptides and amino acids react with carbonyl groups in reducing sugars, to form a Schiff base. This Schiff base then rearranges into Amadori or Heyns products. These intermediates can undergo Strecker degradation by combining with free amino acids to form imines, which are subsequently fragmented to form Strecker aldehydes. Other reactive intermediates of the Maillard reaction are furfural, 5-(hydroxymethyl)furfural (HMF), 4-hydroxy-2,5-dimethyl-3(2H)-furanone (HDMF, furaneol), reductones and acrylamide (AA) [15]. Nevertheless, some of the reaction products, such as acrylamide, are classified as potentially hazardous to human health [16, 17]. For the presence of AA in food, there are allowable limits in various food products according to the EU regulation [18]. However, this regulation does not provide information on the allowable limits for PBMS or similar products. In short, the allowable limits for AA in food vary from 40 (baby food) up to 4000 (coffee substitutes) μg/kg of product. There are no regulations for HMF and HDMF.

There are methods for the determination of AA and other Maillard reaction products in food by commonly known techniques, such as solid-phase extraction (SPE), solid–liquid extraction, purification by Carrez salts combined with liquid chromatography with diode array detection (LC-DAD), liquid chromatography coupled with tandem mass spectrometry (LC-MS/MS). and gas chromatography coupled with mass spectrometry (GC-MS) [19–23]. In the literature, however, there are only few analytical reports on PBMS analysis on possible contaminations. Moreover, they mainly focus on the determination of compounds that could transfer from raw materials to PBMS such as mycotoxins [24, 25], but not on those generated during food production, such as AA. Other investigations in the field of PBMS analysis include the determination of isoflavones by salting out analytes in acetonitrile and subsequent liquid chromatography with Q-Orbitrap detection [24], the profiling of aromatic compounds by headspace solid-phase microextraction (HS-SPME) combined with GC-MS [26] and the evaluation of volatile flavour compounds by solid-phase microextraction (SPME) followed by GC-MS [27]. Nevertheless, the search for new, green alternative methods that take the ecological aspect into account is demanded.

A new generation of solvents, such as deep eutectic solvents (DESs), appear to be an interesting alternative to the commonly used organic solvents as extraction agents with regard to their environmental friendly character [28]. DESs are a mixture of two or more components that are associated via hydrogen bonds and have a lower melting point than their precursors [29]. Recently, natural and biorenewable sources have been utilized to produce natural DESs, which have better environmental and operational properties with low toxicity and high biodegradability. An excellent candidate for the formation of natural DESs is terpenoids, which additionally promote the hydrophobicity of the obtained solvent, allowing the use of eutectic mixtures in the applications that require direct contact with water [30]. Although DESs have found wide application in analytical chemistry for sample preparation, their combination with the most sensitive detection method — mass spectrometry — remains a challenge. This is because DESs typically have high viscosity and low vapour pressure, which have undesirable effects on the analyte ionization and the ion source, which can lead to reduced sensitivity and repeatability [31].

The purpose of this article is to present a novel and robust analytical method for the quantification of AA, HMF and HDMF as particular products of the Maillard reaction in pea-based meat substitutes using DES-based liquid-phase microextraction followed by LC-MS/MS analysis. Since DESs are designable solvents and their properties can be tailored, computational prediction was used to select suitable natural DESs for the effective extraction of processing products from PBMS. To eliminate the potential undesirable effect of a natural DES on the MS detector, back-extraction into the formic acid solution has been utilized. Since the density of the aqueous phase changes during extraction and back-extraction, the inversion of the organic phase was observed, significantly simplifying withdrawing of the desired phase for analysis. To the best of our knowledge, this is a first procedure for the determination of certain products of the Maillard reaction in PBMS using hydrophobic natural DES followed by LC-MS/MS detection.

## Materials and methods

### Chemicals

The standards of analytes, acrylamide (AA) (CAS, 79–06-1; 99% purity), 5-hydroxymethylfurfural (HMF) (CAS, 67–47-0; 99% purity) and 4-hydroxy-2,5-dimethyl-3(2H)-furanone (HDMF, furaneol) (CAS, 3658–77-3; 98% purity), were obtained from Sigma-Aldrich (Merck, Saint Louis, USA). The internal standard of acrylamide-^13^C (AA IS) (CAS, 287399–26-2, 98% purity) was purchased from Toronto Research Chemicals Inc. (Toronto, Canada). LC–MS grade purity methanol (MeOH) was obtained from Supelco Inc. (USA). The ultra-pure water was obtained using the HLP5 system from Hydrolab (Wiślana, Poland). Sodium chloride (CAS, 7647–14-5) and formic acid (CAS, 64–18-6) were purchased from Avantor Performance Materials Poland (Gliwice, Poland). Thymol (CAS, 89–83-8) and eugenol (CAS, 97–53-0) were obtained from Sigma-Aldrich (Merck, Saint Louis, USA).

### Preparation of standard solutions

The stock solutions of the analytes were prepared separately by dissolving standards in MeOH to obtain 500 µg/mL. Working solutions were prepared by diluting stock solutions in ultra-pure water to obtain 50 µg/mL of each analyte. IS solution was prepared by dissolving 1 mg of IS in 1 mL of MeOH and diluted in ultra-pure water to obtain 50 µg/mL. The standard solutions of analytes and IS prepared in this way were used in each step of optimization of sample preparation procedure and selection of chromatographic conditions. Standard solutions were stored at 4 °C in a refrigerator for 3 weeks.

### Samples and matrix sample preparation

Seven pea-based meat substitutes from different brands were purchased from the local market (Gdańsk, Poland). Two products were labelled by the manufacturer as burgers (beef-like and salmon-like), two as meatballs, two as gyros and one as chicken-like pieces. The types and ingredient compositions of real samples were shown in Table S1. The real samples were stored according to the label at 4 °C in the refrigerator for no longer than 4 days. Dried pea was purchased in a local supermarket (Gdańsk, Poland) and served as a matrix for sample preparation procedure and optimization. Before sample preparation, 1 g of dried pea was milled, and 1 mL of ultra-pure water was added. The prepared pea was left to stand for 15 min. For the optimization process, the pea samples were spiked with 20 µL of a mixed solution of analysed compounds (50 µg/mL each) and 20 µL of 50 µg/mL IS solution. The dried pea was stored in a dry and dark place and rehydrated just before usage. Neither pea-based samples nor real samples were heat treated.

### Natural DES preparation

The hydrophobic natural DES was prepared by mixing eugenol and thymol in a molar ratio of 2:1 under stirring at 1000 rpm and heating at a temperature of 70 °C (MS-H280 Pro, Chemland, Stargard, Poland) until a clear liquid was obtained. After cooling, the natural DES was stored at room temperature and used for the DES-based microextraction procedure.

### COSMO-RS calculation

In this research, the ADF COSMO-RS software (provided by SCM, Netherlands) to screen 49 DESs derived from terpenoids was used. The optimization of the molecular geometry for all eutectic mixtures was carried out using the COSMO model for solvation in conjunction with the BVP86/TZVP level of theory. Initially, all the new solvent combinations underwent geometry optimization in a gas-phase environment to identify the most stable conformations. Subsequently, a vibrational analysis was conducted to distinguish the DES conformation that represented the true energy minimum. A comprehensive geometry optimization of the DESs was exclusively performed for the most energetically favourable conformation. Following this optimization process, the logarithmic activity coefficient (ln γ) at infinite dilution under specific conditions, a temperature of 20 °C and a pressure of 101,325 Pa, was calculated. Equation (1) was employed to compute the activity coefficient:

1 $${\text{ln}}\left({\gamma }_{i}\right)=\frac{{\mu }_{i}^{DES}-{\mu }_{i}^{a}}{RT}$$

where:

µ_i_^DES^: chemical potential of analytes in DES.

µ_i_^a^ : chemical potential of pure analytes.

R: universal gas constant (8.314 J/mol).

T: temperature (K).

### Hydrophobic natural DES-based microextraction procedure

**Fig. 1:**
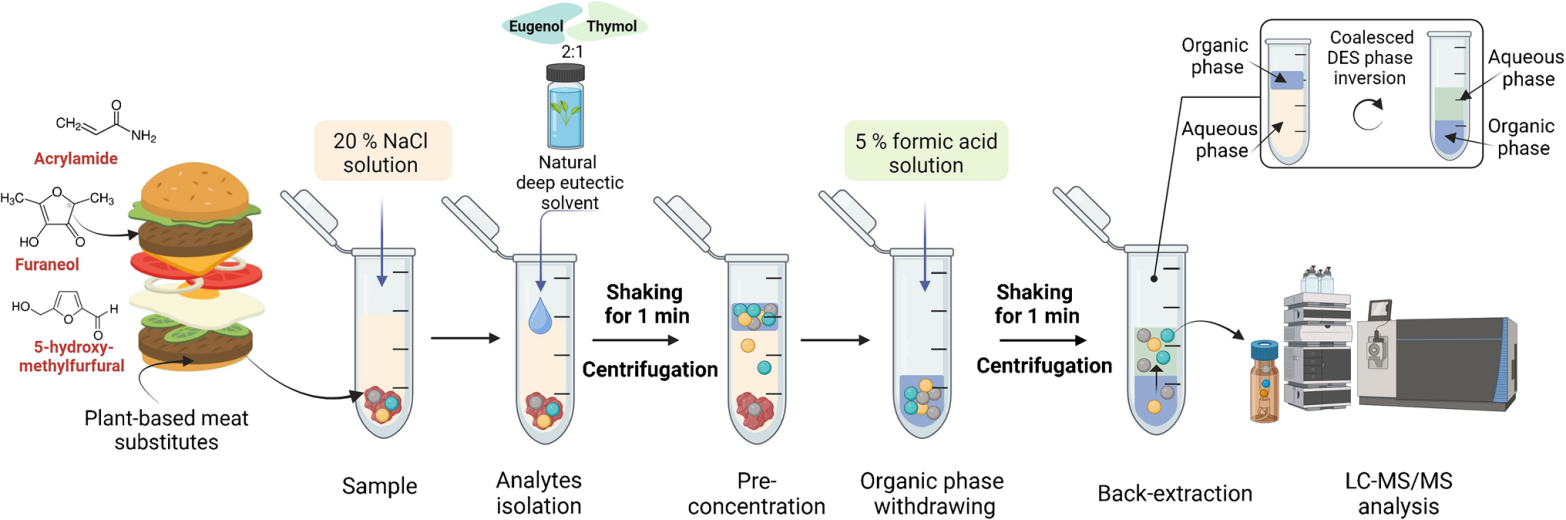
The schematic representation of the hydrophobic natural deep eutectic solvent-based microextraction procedure for the analysis of plant-based meat substitutes

Approximately 150 mg of the real sample or the pea-based matrix sample was placed in a 2-mL Eppendorf tube; 1 mL of a 20% solution of sodium chloride and 300 µL of DES were added (Fig. 1). The sample was mixed for 1 min (1700 rpm, ThermoMixer C, Eppendorf, Hamburg, Germany) and centrifuged for 5 min (6000 rpm, Centrifuge 5084 R, Eppendorf, Hamburg, Germany) to separate the phases. At this stage, the DES phase was coalesced at the top of the vial and was easily withdrawn and transferred into a clean 1.5-mL Eppendorf tube containing 0.5 mL of 5% formic acid solution. Then, the back-extraction step was performed, and the mixture was mixed for 1 min (1700 rpm, ThermoMixer C, Eppendorf, Hamburg, Germany) and centrifuged for 5 min (6000 rpm). At this stage, due to lower density of the formic acid solution in comparison to the organic phase, the coalesced phase inversion was observed, and DES was separated at the bottom of the tube. The aqueous phase (upper one) was collected and filtered through syringe filter (0.22 µm, ø 13 mm, nylon) and placed in the autosampler vial to be analysed by LC-MS/MS. The procedure was applied to real samples, fortified pea-based samples and matrix-matched calibration solutions.

### Calibration curves

Two types of calibration curves were constructed. The solvent-based calibration curves were prepared by diluting stock solutions of analytes in 5% of formic acid solution, to obtain 1, 2, 5, 10, 25 and 50 ng/mL for AA; 10, 25, 50, 100, 500 and 1000 ng/mL for HMF; and 200, 500, 1000, 2500, 7500 and 10000 ng/mL for HDMF. An IS solution was added to each calibration solution to obtain 50 ng/mL.

The matrix-matched calibration curves were prepared by spiking separate pea-based samples (150 mg each) with 20 µL mixed solution of analytes; 0.05, 0.1, 0.25, 0.5, 1.25 and 2.5 µg/mL for AA; 0.5, 1.25, 2.5, 5, 25 and 50 µg/mL for HMF; and 10, 25, 50, 125, 375 and 500 µg/mL for HDMF. Each sample was spiked with 20 µL of 2.5 µg/mL IS solution. The samples were prepared according to the procedure described in “Hydrophobic natural DES-based microextraction procedure”.

The solvent-based and matrix-matched calibration curves of all analytes were compared by Student’s *t*-test to check the influence of the matrix on the results obtained. The *t*-critical value was set at 2.024. For all analytes, the *t*-test results were higher than the critical value; hence, the matrix-matched calibration curves were selected in all cases. In addition, due to the expected content of AA in real samples, a weighted calibration curve was chosen for this analyte to increase precision at lower levels. The weighted calibration curve was performed by using a factor of 1/C, where C was the concentration of the calibration solution. The parameters of the calibration curves were shown in Table 2.

### LC separation and MS/MS conditions

The chromatographic system consisted of an LC (Shimadzu, Japan) equipped with a controller (CBM-20A), a degasser (DGU-20A5R), binary pumps (Nexera X2 LC-30 CE), an autosampler (X2 SIL-30AC) and a column oven (CTO 20AC). The chromatographic system was coupled to the Shimadzu LCMS 8060 MS/MS mass spectrometer with electrospray ionization (ESI). All analyses were performed in the MRM mode with positive ionization. The parameters of selected transitions for all analytes are shown in Table 1.

**Table 1:** Parameters of the chosen MRM transitions

Analyte	Molecular formula	Exact mass (µ)	Precursor ion (M+H)^+^ (m/z)	Product ion (m/z)	Collision energy (V)	Q1 pre-bias (V)	Q3 pre-bias (V)
AA (IS)	^13^C_3_H_5_NO	74	75.1	**58.0**, 45.0	− 17	− 15	− 13
AA	C_3_H_5_NO	71	72.2	**55.1**, 44.0	− 14	− 10	− 22
HMF	C_6_H_6_O_3_	126	127.0	**109.2**, 81.0	− 14	− 13	− 23
HDMF	C_6_H_8_O_3_	128	129.0	**43.1**, 83.1	− 16	− 14	− 18

*bold, quantifier ion

**Fig. 2:**
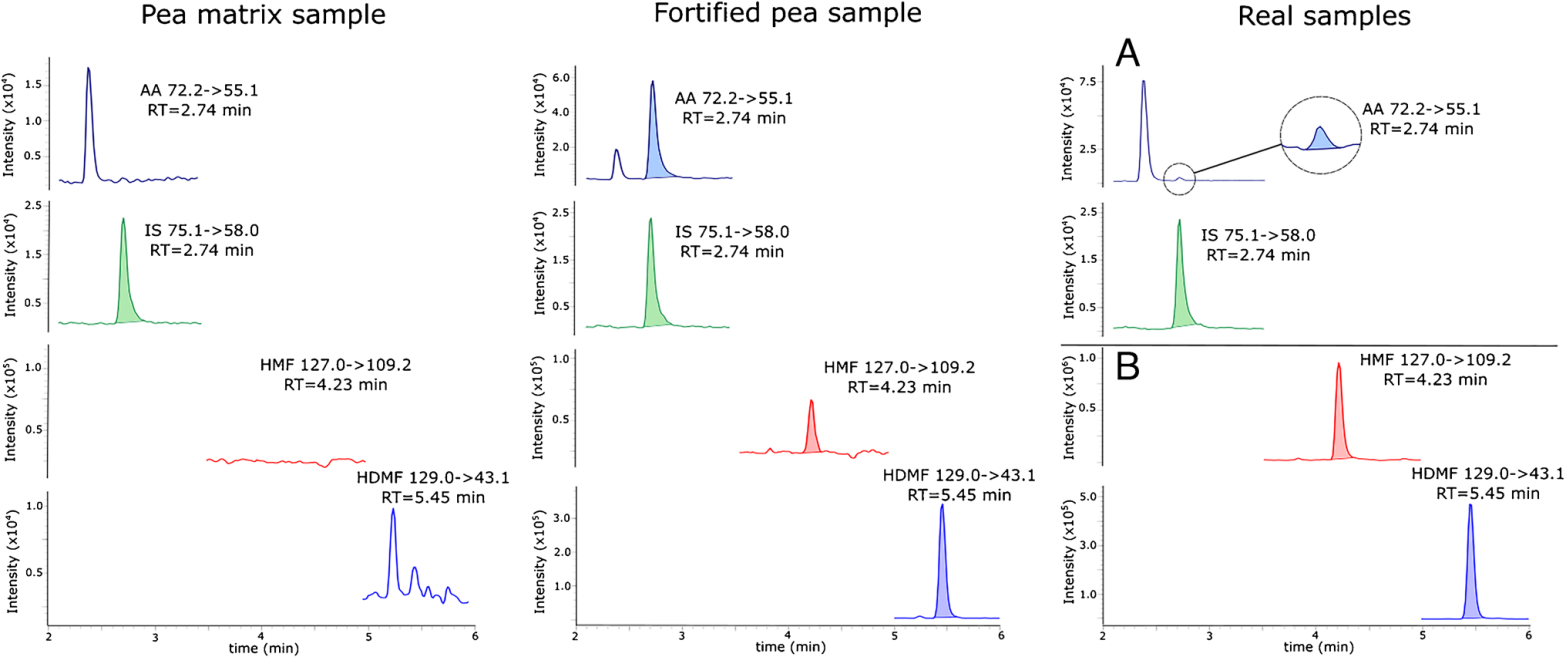
Chromatograms of pea matrix sample fortified with IS (333 ng/g); pea sample fortified with AA (667 ng/g), HMF (667 ng/g), HDMF (67 µg/g) and IS (333 ng/g); a real sample with IS (333 ng/g): A sample No. 8, B sample No. 4

Ion source parameters were as follows: nebulizing gas flow 3 L/min, heating gas flow 10 L/min, interface temperature 300 °C, desolvation line temperature 250 °C, heat block temperature 400 °C and drying gas flow 10 L/min. The system was controlled by LabSolution 5.99 SP2 software, which was used for data acquisition and processing. The separation parameters and analysis of all samples were performed using the Eclipse XDB-C8 column (4.6 × 150 mm, 5 µm), Agilent (USA). The chromatographic conditions were as follows: 0.05% of formic acid in water as a mobile phase component A and methanol as a mobile phase component B. The separation was performed in gradient mode: 0–0.5 min 10% B, 0.5–5 min 60% B, 5–7 min 60% B, 7–11 min, 80% B and 11–16 min 10% B. The flow rate was set to 0.8 mL/min, while the injection volume was kept at 5 µL. The temperature of the column oven was 40 °C. Examples of chromatograms of pea-based matrix sample, fortified pea sample and a real sample were shown in Fig. 2.

## Results and discussion

### DES selection

Before providing of experimental investigation, the computational prediction has been used to select the most suitable composition of DES precursors with the highest affinity to the target analytes. For this purpose, a screening of 45 DESs was conducted owing to the potentially infinite number of possible combinations of hydrogen bond acceptor and hydrogen bond donor. The primary objective was to identify DESs with the greatest ability to dissolve substances, such as AA, HDMF and HMF, thereby improving their extraction efficiency. Binary eutectic mixtures with 1:1 molar ratios were used for the calculations. To ensure the environmentally friendly nature of the novel extraction solvents, substances such as monoterpenoids and carboxylic acids, which can be derived from natural sources such as plants or biomass, were utilized as key ingredients for the DES preparation. The logarithmic activity coefficient (ln γ) at infinite dilution for all analytes was calculated using the COSMO-RS model to assess the suitability of these DESs. The results are depicted in Figure S1 (supplementary materials). The greatest solubilization capacities for AA, HDMF and HMF were demonstrated by the DES formed from the combination of eugenol and thymol. Encouraged by these favourable results, additional calculations were performed for eugenol-to-thymol at different molar ratios (1:2, 2:1, 3:2 and 2:3) in the subsequent stages of the study.

DESs based on eugenol-to-thymol mixtures were checked experimentally with pea-based matrix samples prepared according to the procedure described in the “Hydrophobic natural DES-based microextraction procedure” section to select the most appropriate ratio. The ratios of 1:2 and 2:3 were found to be unstable at room temperature and were excluded from further research. For the remaining ratios, the results of extraction recovery to the target analytes were similar. However, for the DES eugenol-to-thymol at the ratio 2:1, an interesting phenomenon of coalesced DES-phase inversion was observed. Only for this DES precursor ratio at the extraction step the DES phase was collected as an upper phase but at the back-extraction step as a bottom phase. This property significantly simplifies withdrawing of the desired phase, eliminating its contamination.

To gain a more comprehensive understanding and validate the previously mentioned assumptions regarding the interaction between eugenol-to-thymol (2:1) and the analytes, the charge distribution and σ-profile were calculated using 3D surface charge densities [32]. The resulting σ-profiles for both DES and analytes are illustrated in Figure (S2). The significance of compatible σ-profiles between the solvent and extracted compounds has been underscored in prior research. This encompassed the presence of analogous regions, an increase in the σ-profile for one compound and a corresponding decrease for the other, all of which were pivotal for the establishment of strong molecular interactions [33]. These findings strongly indicate that there is substantial potential for the extraction of AA, HDMF and HMF using a DES composed of eugenol and thymol in a 2:1 molar ratio.

### Optimization of the extraction procedure

To optimize the procedure of pea-based meat substitute sample preparation, dried pea was milled and left to stand with water (to 1 g of pea, 1 mL of ultra-pure water was added). The pea-based samples were used in each step of optimization. One-variable-at-the-time (OVAT) was used to select specific parameters. To find the extraction conditions, the following parameters were optimized: sodium chloride solution concentration, DES volume, amount of sample, time of extraction and back-extraction and formic acid solution concentration added during back-extraction. The final procedure was applied to the real samples.

In case of solid-phase samples, it is important to provide effective isolation of the analytes from the solid matrix. Application of electrolytes in high concentrations can support the exaction process due to salting out effect. In the current investigation, sodium chloride was used to enhance the isolation of the target analytes followed by pre-concentration in the DES phase. The concentration of sodium chloride solution varied at 1, 5, 10, 15, 20 and 25%. An increase in extraction recovery was observed at concentrations up to 20%. The difference between 20 and 25% was insignificant; hence, 20% of sodium chloride solution was chosen for further optimization steps.

The amount of sample affected the sensitivity and the measurement uncertainty of the analysis; however, with regard to green chemistry concept, it should be minimized but be sufficient in representativeness as well. Therefore, the sample amount varied from 50 to 250 mg. It was found that the extraction recovery increased with the sample amount up to 150 mg. However, a higher sample amount led to the formation of a solid phase at the interfacial layers.

The DES volume could have an influence on the detection sensitivity. The DES volume was selected among the considered values of 0.1, 0.2, 0.3, 0.4 and 0.5 mL. It was found that the DES volume of 0.3 mL was optimal in both cases — sensitivity and solvent consumption. At lower volumes, proper phase separation was not achieved and at higher volumes the dilution effect predominated.

The effects of both extraction and back-extraction time were investigated. In both cases, 1, 2, 5, 10 and 15 min of mixing time were considered. Neither for the first nor for the second step, the impact of time on the extraction recovery was noticed. One minute of mixing was chosen for both steps, making the sample preparation procedure simple and rapid.

**Fig. 3:**
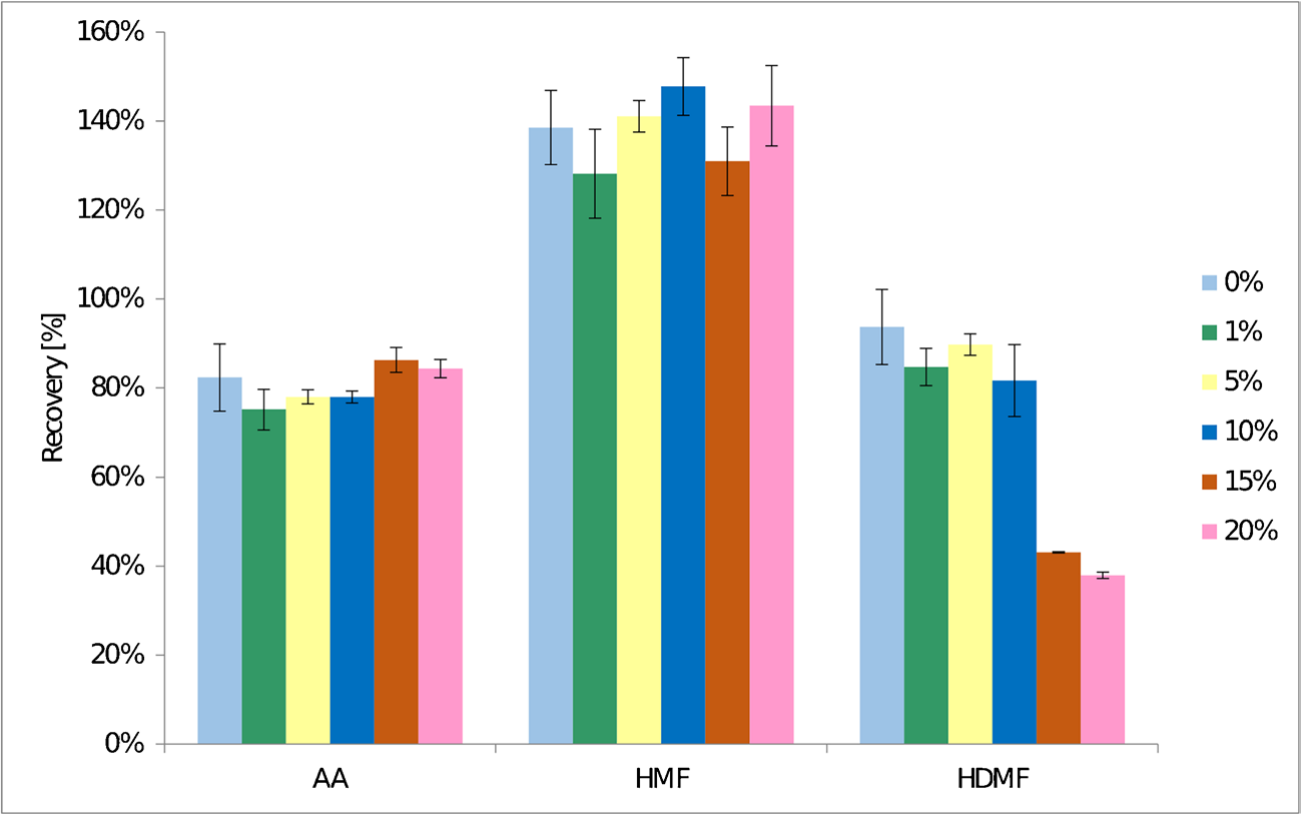
Extraction efficiency for different formic acid concentrations in back-extraction step

To provide a back-extraction, only ultra-pure water was considered. Since the target compounds are well dissolved in water, they could be easily re-extracted into this phase. However, the repeatability of the analytical signal was not achieved. Therefore, formic acid was used to overcome this problem. The application of 1, 5, 10, 15 and 20% of formic acid solution was investigated. For HDMF, a decrease in recovery was observed after formic acid solution addition. For AA and HMF, the tendency was not as clear as for HDMF. However, when formic acid solution was added, an increase in repeatability was observed. The addition of formic acid at 5% concentration led to a decrease in SD from 7.6 to 1.6% for AA, from 8.3 to 3.5% for HMF and from 8.4 to 2.4% for HDMF. In the case of AA, 5% of formic acid solution or more the increase of repeatability was observed. For HMF 5% was optimal, the lowest SD value for HDMF was observed at the highest considered concentration. In this case, the significant decrease in recovery was also observed. The reason of addition of formic acid probably had a positive outcome during ionization of the analytes in the ESI source, since the mobile phase contained also the formic acid. This probably led to better mixing of the sample solvent with the mobile phase and hence increased repeatability. Furthermore, at a formic acid concentration of 20%, the DES phase was separated at the top of the tube that was disadvantageous for the aqueous phase collection. Finally, 5% of formic acid was chosen, as the most suitable for repeatability and recovery behalf. The effect of formic acid solution concentration in back-extraction is shown in Fig. 3.

### Method validation

The performance of the chromatographic method and sample preparation was evaluated. The matrix-matched calibration curves were chosen, due to Student’s *t*-test results. All curves were linear in the selected ranges (*R*^2^ ≥ 0.9952). Due to the expected low concentrations of AA in real samples, the weighted calibration curve was used by applying 1/C factor. The limit of detection (LOD) values were 0.98, 9.8 and 184 ng/mL for AA, HMF and HDMF, respectively, where *LOD* = *3.3S*_*b*_*/a*, where *S*_*b*_ is the standard deviation of the intercept, while *a* is slope. The limit of quantification (LOQ) values was as follows: 3.0 ng/mL for AA, 29 ng/mL for HMF and 553 ng/mL for HDMF. LOQs were calculated according to the formula *LOQ* = *3* × *LOD*. LODs and LOQs were calculated in ng per g of sample and shown in Table 2, next to other parameters of calibration curves for all analytes.

**Table 2:** Parameters of calibration curves

Analyte	Range (ng/mL)	Equation of calibration curve	S_a_	S_b_	LOD (ng/mL)	LOQ (ng/mL)	LOD (ng/g)	LOQ (ng/g)	*R*^2^
AA	1–50	*y* = 0.0367*x* + 0.023*	0.0023	0.011	0.98	3.0	6.5	20	0.9952
HMF	10–1000	*y* = 0.03094*x* – 0.345	0.00020	0.092	9.8	29	65	197	0.9993
HDMF	200–10000	*y* = 0.003209*x* – 0.49	0.000034	0.18	184	553	1229	3688	0.9982

*weighted calibration curve 1/C

Precision and accuracy were checked by spiking pea-based matrix samples with three levels of analyte concentration. Each spiking level was prepared with three replicates. The spiked pea-based matrix samples were prepared according to the protocol described in the “Hydrophobic natural DES-based microextraction procedure” section. The recoveries, SDs and coefficient of variation (CV) values were shown in Table 3. The results obtained with developed method using DES followed by LC-MS/MS allow successful analysis of AA, HMF and HDMF in pea-based meat substitutes.

**Table 3:** Precision and accuracy data for spiked pea-based samples prepared following the optimized microextraction procedure

Analyte	Spiking level (ng/mL)	Recovery ± SD (ng/mL) (%)	CV (%)
AA	3	3.62 ± 0.18 (120)	4.9
	10	9.34 ± 0.17 (93)	1.8
	25	27.0 ± 1.0 (108)	3.8
HMF	30	29.66 ± 0.72 (99)	2.4
	60	56.9 ± 1.4 (90)	2.6
	90	99.7 ± 3.3 (111)	3.3
HDMF	350	369.3 ± 7.8 (106)	2.1
	1000	830 ± 10 (83)	1.2
	5000	4430 ± 150 (89)	3.4

### Real sample analysis

Seven pea-based meat substitutes were prepared according to the protocol described in the “Hydrophobic natural DES-based microextraction procedure” section and analysed by LC-MS/MS. Each sample was analysed in triplicate. The results calculated to ng/g were shown in Table 4. Due to the high concentration of HMF and HDMF, the extracts gained from samples 1, 3, 4 and 5 were diluted ten times in 5% of formic acid solution and re-analysed. Sample 1 needed to be diluted 100 times to be analysed due to high concentration of HDMF. The concentration of AA was below the LOD in five samples and detected, but below LOQ, in two samples. HMF was detected in five samples. HDMF was detected in all samples, but in three samples, it was not quantified. Examples of real sample chromatograms were shown in Fig. 2.

**Table 4:** Results of analysis of real samples

Sample	Type of sample	AA ± SD (ng/g)	HMF ± SD (ng/g)	HDMF ± SD (ng/g)
1	Beef-like burger	< LOD	1424 ± 170*	286700 ± 28000**
2	Salmon-like burger	< LOD	413 ± 42	< LOQ
3	Plant-based meatballs	< LOD	1219 ± 130*	25300 ± 4800*
4	Plant-based meatballs	< LOD	5291 ± 540*	4775 ± 430
5	Plant-based gyros	7.7 ± 1.9	2566 ± 330*	< LOQ
6	Plant-based gyros	< LOD	< LOD	8406 ± 1000
7	Chicken-like pieces	17.8 ± 2.8	< LOD	< LOD

*original extract diluted ten times in 5% FA and re-analysed

**original extract diluted 100 times in 5% FA and re-analysed

### Evaluation of the practicability of the developed procedure

The blue applicability grade index (BAGI) [34] was used to evaluate the practicality of the developed procedure. This tool includes parameters such as the type of analysis, the number of analytes determined, the sample throughput, the type of reagents, materials used for the procedure, the steps of sample preparation, type of sample preparation, the instrumentation required, the possibility of automation and the sample amount. It should be noted that the developed procedure is miniaturized and requires only 150 mg of the sample for the simultaneous determination of three processing compounds using common reagents available in the laboratory. Even if the synthesis of DES is required, it assumes simple mixing of two available components. Sample preparation is rapid (less than 15 min) and LC-MS/MS analysis takes 18 min. Moreover, since the ThermoMixer was used for mixing, it is possible to mix 24 samples (1.5 mL Eppendorf tubes) simultaneously. The developed procedure is used for solid-phase food samples; hence, it is performed manually without using any kind of automated equipment or flow methods. The calculation using the BAGI software resulted in a total score of 65.0, which shows a good applicability potential of the developed procedure Figure S3*.*

## Conclusions

In this paper, hydrophobic natural DES was used for the first time for the determination of Maillard reaction products in PBMS. The sample preparation involves the use of an innovative green solvent, which is more environmental friendly than the commonly used organic solvents. Moreover, the application of back-extraction is a workaround for the use of DES in sample preparation followed by LC-MS/MS analysis, which is rarely to be found or presented. Nevertheless, the limitation of the procedure is that the extra step is needed to allow the sample to be analysed by MS/MS. The method was validated for the determination of AA, HMF and HDMF and successfully applied to real samples analysis. Due to large concentrations of HMF and HDMF in analysed products, further dilution of the extracts was required, gained by the presented sample preparation procedure. However, analysis of PBMS and its potential contamination profiling is needed, to monitor this novel type of food. Further studies should focus on the analysis of a wide range of commercially available products derived from different plants, mainly soy, wheat or chickpea.

### Supplementary Information

Below is the link to the electronic supplementary material.

Supplementary file1 (DOCX 780 KB)
