# Supplementary material for: Plant-based meat substitute analysis using microextraction with deep eutectic solvent followed by LC-MS/MS to determine acrylamide, 5-hydroxymethylfurfural and furaneol
Source: Anal Bioanal Chem. 2023 Dec 21;416(5):1117–26. doi: 10.1007/s00216-023-05107-6 (PMC10850178; doi:10.1007/s00216-023-05107-6)
Supplement: Supplementary file 1 — Supplementary file1 (DOCX 780 KB) [file 216_2023_5107_MOESM1_ESM.docx]

**Plant-based meat substitutes analysis using microextraction with deep eutectic solvent followed by LC-MS/MS to determine acrylamide, 5-hydroxymethylfurfural and furaneol**

Dominika Osiecka^1^, Christina Vakh^1,2^ , Patrycja Makoś-Chełstowska^3^, Paweł Kubica^1,^*

*^1^Department of Analytical Chemistry, Faculty of Chemistry, Gdańsk University of Technology, 11/12*

*G. Narutowicza Street, 80-233 Gdańsk, Poland*

*^2^EcoTech Center, Gdańsk University of Technology, 11/12 G. Narutowicza Street, 80-233 Gdańsk, Poland*

*^3^Department of Process Engineering and Chemical Technology, Faculty of Chemistry, Gdańsk University of Technology, 11/12 G. Narutowicza Street, 80-233 Gdańsk, Poland*

**Corresponding author’s e-mail:** pawel.kubica@pg.edu.pl

**Supplementary Materials**

**Structural and physical properties of DES**

Structural characterization of DES was studied using ATR-FTIR spectroscopy based on Bruker Tensor 27 spectrometer (Bruker, USA) equipped with an ATR accessory and OPUS software (Bruker, USA). The experimental parameters employed were as follows: a spectral range spanning from 4000 to 600 cm^−1^, 256 scans each for background and sample, a resolution of 4.5 cm^−1^, and a slit width of 0.5 cm. To acquire the ^1^H NMR and ^13^C NMR spectra of the DES, we weighed 20 mg of the DES and mixed it with 0.7 mL of chloroform-d1. The measurements were conducted at a temperature of 20 °C using the Bruker Avance III HD 400 MHz instrument (Bruker, Billerica, MA, USA). The dynamic viscosity and density of the DES were assessed over a temperature range spanning from 20 to 50°C. These measurements were carried out using a BROOKFIELD LVDV-II+ viscometer (Labo-Plus, Poland) and a DMA 4500 M density meter (Anton Paar, Poland), respectively.

**COSMO-RS calculation**

Screening of 45 Deep Eutectic Solvents (DESs) was conducted owing to the potentially infinite number of possible combinations of HBA and HBD. The primary objective was to identify DESs with the greatest ability to dissolve substances, such as AA, HDMF, and HMF, thereby enhancing their extraction efficiency. Binary eutectic complexes with 1:1 molar ratios were employed for calculations. To ensure the environmentally friendly nature of the novel extraction solvents, substances such as monoterpenes and carboxylic acids, which can be derived from natural sources such as plants or biomass, were utilized as key ingredients in DES preparation. The logarithmic activity coefficient (ln γ) at infinite dilution for all analytes was calculated using COSMO-RS model to assess the suitability of these DESs. The results are depicted in Figure S1, with lower values (more negative values) indicating a stronger affinity of the DESs for the analytes. The results indicate that the highest affinity of DESs for all analytes was observed in eutectic complexes containing thymol and eugenol in their structure. Both substances, when combined with carboxylic acids, exhibited high affinities for all analytes. Nevertheless, the greatest solubilization capacities for AA, HDMF, and HMF were demonstrated by the DES formed from the combination of eugenol and thymol. DESs composed of eucalyptol, carvone, camphor, and menthol, either in combination with carboxylic acids or other monoterpenes, displayed the lowest affinities for all analytes. This reduced affinity can be attributed to the limited number of hydrogen bond donor groups capable of forming strong noncovalent bonds with the analytes. In the case of eugenol:thymol (1:1), both components contain an -OH group, which can engage in strong hydrogen bonds with the carbonyl group (=O) and oxygen atom in the furan ring (-O-). For AA, HDMF, and HMF, the logarithmic activity coefficients were -1.3, -0.96, -5.1, and -1.7 for AA, HDMF, and HMF, respectively. Encouraged by these favorable results, additional calculations were performed for eugenol:thymol at different molar ratios (1:2, 2:1, 3:2, and 2:3) in the subsequent stages of the study. The results revealed that as the eugenol content in DES increased, the analyte attachment capacity also increased. At a molar ratio of 2:1, the ln γ values were -1.7, -1.1, -5.8, and -2.0 for AA, HDMF, and HMF, respectively. This phenomenon can be attributed to the presence of a methoxy group (-O-CH₃), which can form additional noncovalent bonds with the amine, hydroxyl, and alkyl groups present in the structure of the analytes.

To gain a more comprehensive understanding and validate the previously mentioned assumptions regarding the interaction between eugenol:thymol (2:1) and the analytes, the charge distribution and σ-profile were calculated using 3D surface charge densities [1]. The resulting σ-profiles for both DES and analytes are illustrated in Figure S2. The σ-profile graph was segmented into three distinct interaction regions: a non-polar region encompassing the range of −0.0084 e/A2 > σ < 0.0084 e/A2, a hydrogen bond donor region (σ < −0.0084 e/A2), and a hydrogen bond acceptor region (σ > 0.0084 e/A2) [2–5]. The potential of the studied components to engage in robust hydrogen bonding is underscored by both the hydrogen bond donor and acceptor regions. Remarkably, similar results were observed for all analytes and DES. The most prominent peak was found in the nonpolar region, whereas smaller peaks were evident in the hydrogen bond donor and acceptor regions. This suggests that both the analytes and DES components can be classified as hydrogen bond donors and acceptors. Furthermore, the significance of compatible σ-profiles between the solvent and extracted compounds has been underscored in prior research. This encompassed the presence of analogous regions, an increase in the σ-profile for one compound, and a corresponding decrease for the other, all of which were pivotal for the establishment of strong molecular interactions [6]. These findings strongly indicate that there is substantial potential for the extraction of AA, HDMF, and HMF using a deep eutectic solvent composed of eugenol and thymol in a 2:1 molar ratio.


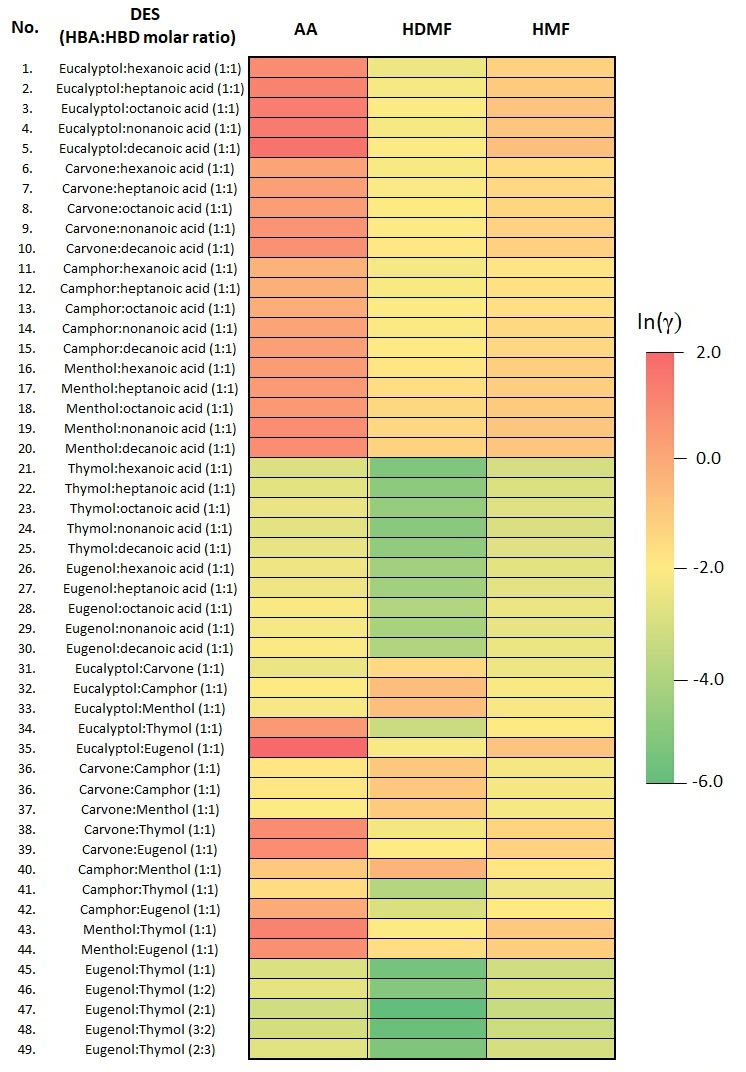


Figure S1 Matrix of calculated activity coefficients at infinite dilution of analytes in various DESs


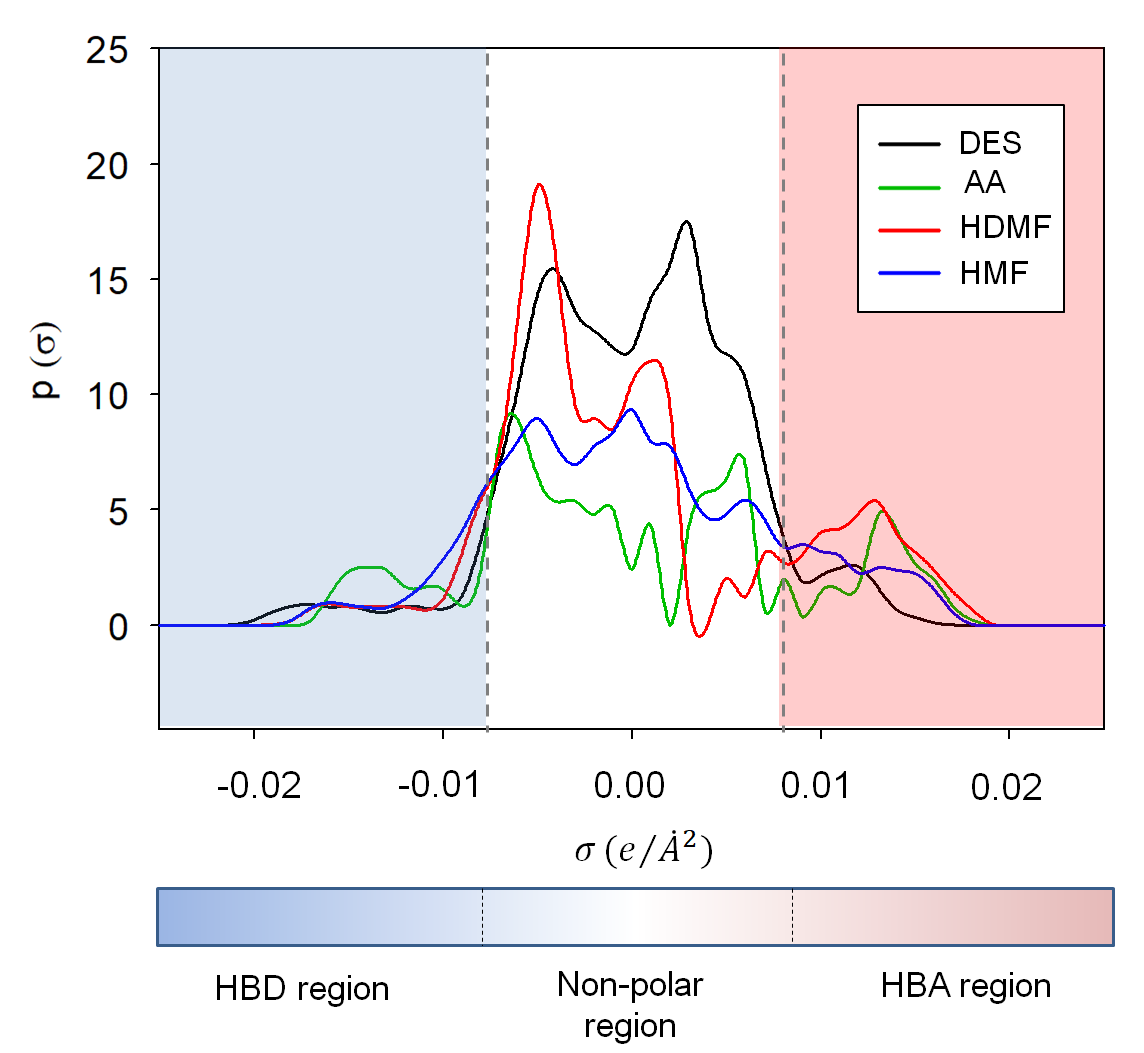


Figure S2 σ-profiles of DES (eugenol:thymol 2:1), and anlaytes (AA, HDMF and HMF)

Table S1 Types and compositions of ingredients of analysed real samples

| Number of sample | Type | Ingredients |
| --- | --- | --- |
| 1 | Beef-like Burger | Water, pea protein (22%), canola oil, natural aromas, potato starch, stabilizer: methylcellulose, bamboo fiber, dried spirit vinegar, barley malt extract, dried onion, dried garlic, dried beetroot, salt, iron, B_12_ vitamin |
| 2 | Salmon-like burger | Water, pea protein (23%), canola oil, natural aromas, rice starch, stabilizer: methylcellulose, dried garlic, bamboo fiber, spices, dried spirit vinegar, dried beetroot, salt, dried ramson (0.04%), barley malt extract, iron, B_12_ vitamin, smoke aroma |
| 3 | Plant-based meatballs | Water, pea protein (23%), canola oil, natural aromas, pumpkin seeds protein, stabilizer: methylcellulose, potato starch, dried red onion (0.9%), dried white onion, dried spirit vinegar, dried thyme (0.5%), salt, barley malt extract, dried beetroot, iron, B_12_ vitamin |
| 4 | Plant-based meatballs | Water, fried onion (onion 22%, canola oil), texturized pea protein (23%), canola oil, spices mixture [aromas, spices (including onion, garlic), sugar, salt], thickening agent: methylcellulose, cellulose; caramelized sugar, dried milled beetroot, spices extract, aroma, salt, pepper |
| 5 | Plant-based gyros | Water, extruded pea protein 33.2%, canola oil, wheat protein 5.5%, spices, salt, sugars (sugar, caramelized sugar), aromas |
| 6 | Plant-based gyros | Water, texturized pea protein, canola oil, natural aromas, spices, dried red sweet pepper, salt |
| 7 | Chicken-like pieces | Water, pea protein 33%, canola oil, pea fiber, spices, salt, marjoram, natural aromas, B_12_ vitamin |


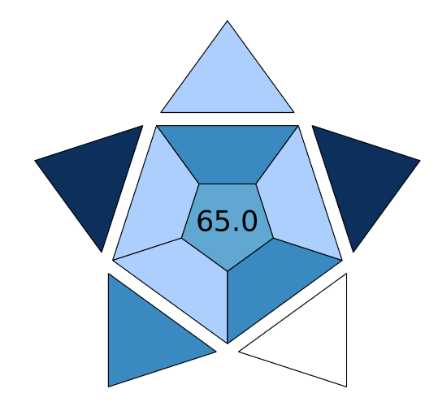


Figure S3 The BAGI index pictogram for the developed procedure

References

1. Ozturk B, Gonzalez-Miquel M (2019) Alkanediol-based deep eutectic solvents for isolation of terpenoids from citrus essential oil: Experimental evaluation and COSMO-RS studies. Sep Purif Technol 227:115707. https://doi.org/10.1016/J.SEPPUR.2019.115707

2. Słupek E, Makoś-Chełstowska P, Gębicki J (2021) Removal of siloxanes from model biogas by means of deep eutectic solvents in absorption process. Materials 14:1–20. https://doi.org/10.3390/ma14020241

3. Makoś-Chełstowska P, Słupek E, Małachowska A (2022) Superhydrophobic sponges based on green deep eutectic solvents for spill oil removal from water. J Hazard Mater 425:. https://doi.org/10.1016/j.jhazmat.2021.127972

4. Ojeda RM, Llovell F (2018) Soft-SAFT Transferable Molecular Models for the Description of Gas Solubility in Eutectic Ammonium Salt-Based Solvents. J Chem Eng Data 63:2599–2612. https://doi.org/10.1021/acs.jced.7b01103

5. Chu Y, He X (2019) MoDoop: An Automated Computational Approach for COSMO-RS Prediction of Biopolymer Solubilities in Ionic Liquids. ACS Omega 4:2337–2343. https://doi.org/10.1021/acsomega.8b03255

6. Chemat F, Anjum H, Shariff AM, Kumar P, Murugesan T (2016) Thermal and physical properties of (Choline chloride + urea + l-arginine) deep eutectic solvents. J Mol Liq 218:301–308. https://doi.org/10.1016/J.MOLLIQ.2016.02.062
